# Supplementary material for: High Purity Struvite Recovery from Hydrothermally-Treated Sludge Supernatant Using Magnetic Zirconia Adsorbent
Source: Int J Environ Res Public Health. 2022 Oct 13;19(20):13156. doi: 10.3390/ijerph192013156 (PMC9602817; doi:10.3390/ijerph192013156)
Supplement: Supplementary file 1 [file ijerph-19-13156-s001.zip › ijerph-1947043-supplementary.pdf]

Supplementary files

High Purity Struvite Recovery from Hydrothermally Treated Sludge Supernatant Using Magnetic Zirconia Adsorbent

Zhe Wang, Shuai Guan, Yajuan Wang, Wen Li, Ke Shi, Jiake Li and Zhiqiang Xu *

State Key Laboratory of Eco-hydraulics in Northwest Arid Region of China, Xi'an University of Technology, No. 5 South Jinhua Road, Xi'an 710048, China

* Correspondence: zqxuxaut@163.com (Z.X.)

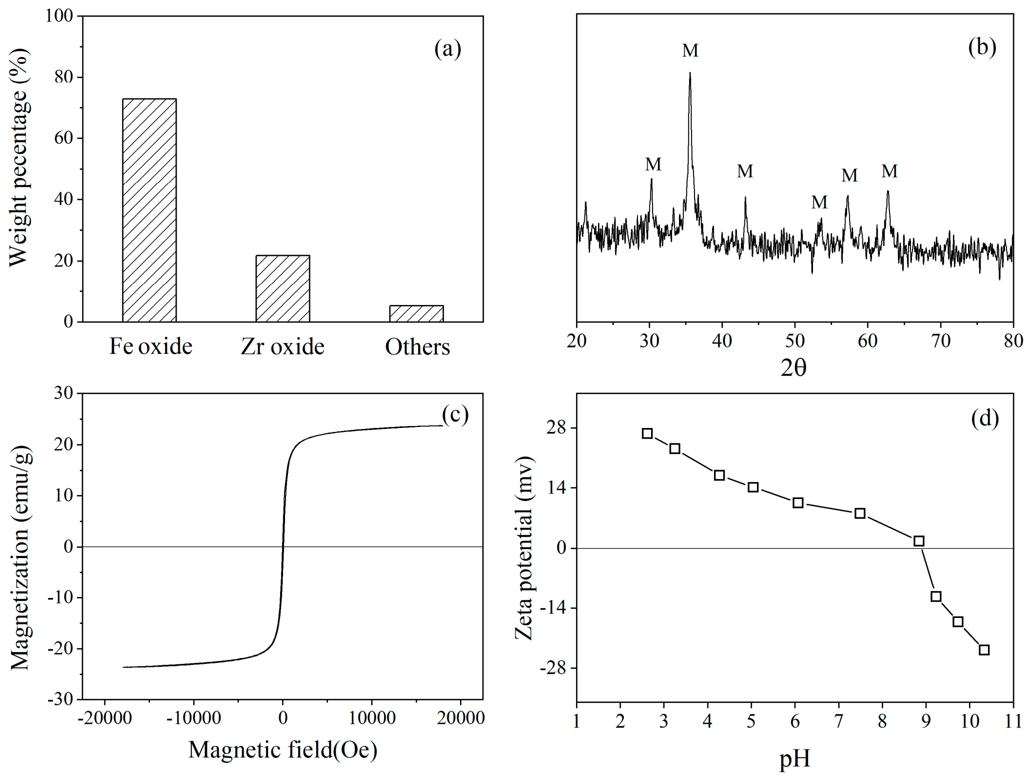

Figure. S1 Chemical composition (a), X-ray diffraction pattern (b), magnetization curve (c) and Zeta potential variation under different pH (d) of the MZ adsorbent. M in Fig. S1b indicated the crystal phase of magnetite.

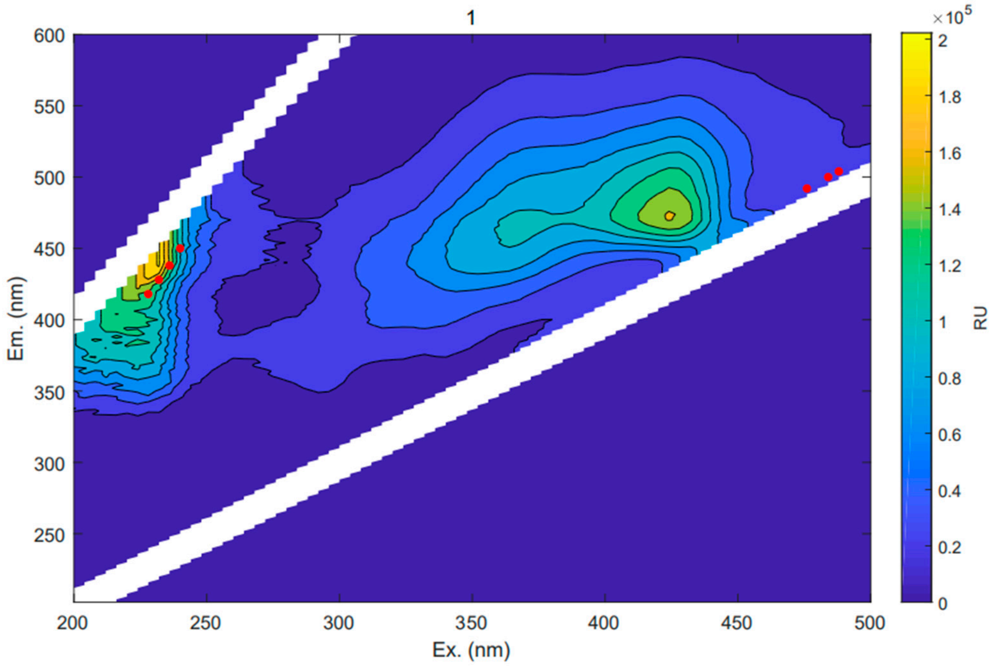

Figure. S2 Three-dimensional fluorescence distribution of the HTSS
